# Recent advances in understanding and treating chronic granulomatous disease

**DOI:** 10.12688/f1000research.11789.1

**Published:** 2017-08-11

**Authors:** Andrew Gennery

**Affiliations:** 1 Paediatric Immunology and Haematopoietic Stem Cell Transplantation, Great North Childrens’ Hospital, Newcastle upon Tyne, UK; 2 Primary Immunodeficiency Group, Institute of Cellular Medicine, Newcastle University, Newcastle upon Tyne, UK

**Keywords:** chronic granulomatous disease, haematopoietic stem cell transplantation, gene therapy, inflammatory bowel disease, X-linked carrier disease

## Abstract

A number of recent advances have been made in the epidemiology and treatment of chronic granulomatous disease. Several reports from developing regions describe the presentations and progress of local populations, highlighting complications due to Bacillus Calmette–Guérin vaccination. A number of new reports describe complications of chronic granulomatous disease in adult patients, as more survivors reach adulthood. The complications experienced by X-linked carriers are particularly highlighted in three new reports, confirming that infection and inflammatory or autoimmune conditions are more common and severe than previously recognised. Finally, definitive treatment with haematopoietic stem cell transplantation and gene therapy is reviewed.

## Introduction

Chronic granulomatous disease (CGD) results from an inherited defect in one of six components of the phagocyte nicotinamide adenine dinucleotide phosphate (NADPH) oxidase enzyme complex, responsible for generating reactive oxygen species (ROS) released during the phagocyte respiratory burst to eliminate ingested micro-organisms. Two membrane-bound cytochrome heterodimer components—gp91 ^phox^ and p22 ^phox^ (encoded by *CYBB* and CYBA respectively)—embedded within the membrane of lysosomes come together with the four cytosolic components—p47 ^phox^, p67 ^phox^, p40 ^phox^, and *RAC2* (encoded by *NCF1*, *NCF2*, *NCF4* and *RAC2* respectively)—to activate NADPH to produce hydrogen peroxide. Transfer of electrons from the NADPH complex to molecular oxygen creates a negative charge within the phago-lysosome. This enables influx of potassium through calcium-activated potassium channels. Thus, microbial killing likely occurs through direct action of ROS upon microbes as well as super oxidase-mediated activation of additional killing pathways.

In out-bred populations, X-linked disease due to mutations in *CYBB* is most common. There are numerous clinical manifestations, but the hallmark is severe invasive, bacterial or fungal infection, frequently due to catalase-positive organisms or *Aspergillus* species. However, inflammation plays an important role in the pathogenesis and sequalae of the disease. Much information regarding the epidemiology, clinical manifestations, treatment approaches and outcome of patients with CGD has been gathered over the years, since the first description by Robert Good in 1957 ^1^. This review will focus on newly published information over the past couple of years.

## Epidemiology

### Chronic granulomatous disease in developing regions

To date, most information regarding presentation and outcome of patients with CGD has come from developed countries ^2– 9^. However, the genetic diversity and presentation of primary immunodeficiencies are dependent on the specific population and environmental influence respectively, and published information to date does not reflect the disease in other geographical areas of the world. Recent publications from India, Central and Latin America, Africa and the Middle East and China have given a wider perspective on the disease. Although ascertainment is incomplete and shows bias, some conclusions can be drawn. The Chinese cohort of 48 patients shows some interesting trends ^10^, confirming observations from a previous Chinese study ^11^. Most patients had X-linked CGD, confirming the predominance of X-linked disease worldwide in non-consanguineous populations. Most diagnoses were after 2010 and presumably this was due to greater awareness of the disease. All were diagnosed in early childhood. There was a high mortality (22%), and deaths were due to infection. Complications due to Bacillus Calmette–Guérin (BCG) vaccination were particularly prevalent. Inflammatory complications were not reported, and there were no adults in the cohort. A number of mutations were reported, although in previous series from China the accuracy of the data has been questioned ^11, 12^.

Two Indian CGD patient cohorts have been reported recently. In one, there was a similar incidence of X-linked and autosomal recessive (AR) CGD ^13^. Infections were the predominating clinical problem, although significant BCG infections were rare. All patients were diagnosed in early childhood and there was a significant mortality of 29%. Most cases were diagnosed in the 3 years before the report was published. The second cohort compared 21 patients with the same genetic deletion in p47 ^phox^ (a two-base pair GT dinucleotide deletion at the beginning of exon 2 in *NCF1*, the most common defect in p47 ^phox^-defective CGD). All patients were diagnosed in early childhood, and infection was the most significant clinical problem. Mortality was high at 24% ^14^.

Three Mediterranean cohorts—from Egypt, Tunisia and Israel—have been published recently. The Egyptian cohort of 28 patients showed a predominance of AR-CGD (82%), mainly due to mutations in *CYBA* ^15^. Whilst infection was the most common presentation, BCG complications were less common and inflammatory complications were reported in this paediatric cohort. Additionally, the study showed the value of intracellular staining of NADPH components using specific monoclonal antibodies followed by flow cytometric analysis in aiding the diagnosis. The Tunisian study reported on 11 patients from contiguous regions within western Tunisia, with a unique mutation in *NCF2*, and demonstrated a founder effect ^16^. All patients presented in childhood with infection, a high incidence of tuberculosis and a mortality of 80%. Other CGD cases in the country demonstrate different forms of AR-CGD. This study highlights the importance of local knowledge of a disease; similar founder effects have been found in populations in Oman ^17^ and Korea ^18^ and in Kavkazi Jews ^19^. An Israeli study of 84 patients looked at a mixed population of Jewish and Arab patients ^20^. X-linked disease was more common in the Jewish populations, and AR-CGD was found predominantly in consanguineous Arab and Jewish populations. Severity of disease was related to the degree of residual oxidative burst, confirming a previous report ^21^. Most patients presented early in childhood with infection; mycobacterial infection did not predominate in this series. Granulomatous inflammation was commonly reported. A quarter of patients received haematopoietic stem cell transplantation. Overall survival was good, and mortality since 2008 was 2%, reflecting improvements in medical management.

A Mexican series describes a cohort of 27 patients with CGD, amongst a larger primary immunodeficiency disorder population ^22^. The mortality was 33%. Disappointingly, survival was no different in patients diagnosed before or after 2001, and it dropped dramatically after 10 years of age. The Latin American Society for Immunodeficiencies studied a cohort of 71 patients from five countries, including Mexico ^23^. X-linked CGD was most commonly represented (75%), followed by defects in p47 ^phox^ (23%). Pulmonary infectious presentation was most common; BCG complications occurred in 30% (predominantly those with X-linked disease). Some patients had undergone haematopoietic stem cell transplantation, whilst others were receiving prophylaxis. There were no recorded deaths in the cohort during the study period.

Several observations can be made from these studies. Firstly, they confirm that whilst X-linked CGD is found most commonly in out-bred populations, AR disease is more common in areas with high consanguinity, and specific founder effects are evident in particular populations. Secondly, infectious issues predominate, in a pattern and with micro-organisms that are commonly seen in developed countries. The exceptions to this—tuberculosis or infectious complications secondary to BCG vaccination—make up a significant proportion of the infections reported in these studies. The high morbidity associated with BCG vaccination suggests that screening for CGD could be considered in these populations before BCG is administered. Mycobacterial infection is a recognised complication of CGD, although in a published large multi-centre series, the isolates were BCG or *Mycobacterium tuberculosis* rather than *Mycobacterium leperae* or environmental mycobacteria ^24^. In this survey, 24% of patients presented solely with mycobacterial infection. Thirdly, whilst infections were a predominant clinical problem, inflammatory complications were rarely reported. Whether this was due to ascertainment bias, a lack of recognition or a high, infectious early mortality is not clear. Despite tremendous advances in medical care within developed countries, CGD continues to have a high mortality in less advantaged countries. Greater awareness amongst physicians in these regions may help with earlier recognition and treatment, and registry data would further map the genetic pattern and clinical complications.

### Chronic granulomatous disease in adults

A number of new reports have given further information on adult patients with CGD. Adult patients fall into three categories: those diagnosed as children and surviving to adulthood ^25^, those who are symptomatic in childhood but are diagnosed only as adults ^26^ and those with mild disease who present for the first time as adults ^26^. A survey of the French CGD cohort reported on 80 adult patients with CGD who were all diagnosed by the age of 16 years and thus gives insight into the clinical course of patients in a developed healthcare system, diagnosed in the modern era when chemo-prophylaxis with antibacterial and antifungal agents was employed. Several important findings were presented. Firstly, despite appropriate prophylaxis, infections occurred every 3.75 patient-years. The frequency of infections in adults was no different to those in children. Furthermore, 10 patients (12.5%) died in adulthood, almost all from infectious causes, confirming that infection is the leading cause of death in CGD, even in adults receiving appropriate prophylaxis. The most common pathogens remained *Staphylococcus aureus* and *Aspergillus* species. Secondly, inflammatory and autoimmune complications were extremely common, affecting almost 90% of patients. These complications were as common in adulthood as in childhood, but the first event occurred at a later age than the first infectious presentation, perhaps explaining why inflammatory and autoimmune manifestations were under-reported in cohorts from developing regions. Thirdly, there was a significant burden of chronic disease in the adult cohort, including short stature, particularly in those receiving corticosteroids in adolescence. Additional findings included low body weight, chronic dyspnoea, often associated with radiological changes, and gastrointestinal complications. The findings in this report emphasise that the CGD disease burden transfers and may deteriorate during adulthood and, though not explicitly stated, likely negatively impacts quality of life. Furthermore, despite modern management, there is an ongoing mortality associated with the disease.

A separately published study of the same adult French CGD cohort specifically examined respiratory complications ^27^. Nine (13%) of 67 patients died, predominantly from *Aspergillus* species infection. Infective or inflammatory respiratory complications were common and were found more frequently than in childhood, despite appropriate antimicrobial chemo-prophylaxis. Treatment of these complications was complicated and often was prolonged for more than 6 months and frequently involved lengthy hospitalisation and invasive, sometimes multiple therapies. Inflammatory respiratory complications occurred in more than 25% of patients, and non-infectious complications were more likely to be persistent. Several issues are raised by this study. It gives insight into the natural history of CGD, modified through therapeutic intervention, and particularly highlights patients presenting with less severe disease, modified by genetics or environmental factors or both. Additionally, it raises awareness of risks and benefits of earlier intervention in the disease course. Finally, it demonstrates the challenge to adult physicians, managing patients with potentially unfamiliar “paediatric” diseases.

Two reports describe patients presenting with AR-CGD in adulthood. Colin de Verdière and colleagues describe two unrelated adults who presented with respiratory symptoms due to unusual infectious micro-organisms ( *Aspergillus fumigatus* and *Pantoea* species in one and *Nocardia* species in the other) ^28^. Both had earlier presented in early adulthood with inflammatory bowel disease. A report from Iran describes a 40-year-old man with a history of granulomatous lung lesions and recurrent skin and soft tissue abscesses who presented with respiratory symptoms ^29^. Histological examination demonstrated lymphocytic bronchiolitis, and subsequently a diagnosis of CGD was confirmed.

These cases demonstrate that vigilance is required when confronted with adult patients presenting with unusual infections, especially if they are due to catalase-positive micro-organisms which are specifically associated with CGD ^30^ or if other infectious or inflammatory features are evident in the history; it is likely that CGD in adults is more common than currently recognised.

Whilst defective NADPH function is generally associated with unfavourable sequelae, emerging evidence suggests that mutations in *CYBB* and *NCF1* may play a protective role in inflammatory arterial disease. Enhanced production of pro-inflammatory cytokines by defective myeloid cells in CGD patients contributes to the exuberant inflammatory response in response to, or independent of, infection. Excess inflammation has also been implicated in the pathogenesis of cardiovascular disease and atherosclerosis, particularly associated with increased ROS generated by the NADPH oxidase (NOX) protein family, NOX2, NOX1, and NOX4. NOX2 contributes to the vascular endothelial cell response to nitric oxide and has increased expression in the atherosclerotic plaque in inflammatory vascular disease. NOX2 is also expressed in platelets, inducing platelet aggregation and thrombosis. Reduced NOX activity may diminish vascular inflammation and platelet aggregation and so decrease susceptibility to atherosclerosis.

A recent study provides evidence that reduced NOX activity in CGD patients diminishes the risk of atherosclerosis ^31^. In a study of 41 patients with X-linked or p47 ^phox^-deficient CGD, who had increased risk factors for arterial inflammation, including hypertension, oxidised low-density lipoprotein, and low high-density lipoprotein, a significantly lower internal carotid artery wall volume was found compared with healthy control subjects although coronary arterial calcification was similar in the two groups ^31^.

## Inflammatory bowel disease

New information has recently been published in relation to clinical presentation of patients with CGD and, in particular, in relation to gastrointestinal symptoms. A study of 93 patients followed at the National Institutes of Health (NIH) compared patients with inflammatory bowel disease with those without disease, specifically looking for associations with previously published inflammatory bowel disease risk single nucleotide polymorphism genotypes ^32^. Common inflammatory bowel disease risk alleles were associated with CGD inflammatory bowel disease, but the association was less strong than for that in paediatric patients with Crohn’s disease, confirming that reduced superoxide production is an independent risk factor for development of inflammatory bowel disease.

A small study of eight asymptomatic patients sought to evaluate whether faecal calprotectin predicted sub-clinical inflammatory bowel disease. Only one patient had raised values, who subsequently developed colitis ^33^. Further studies are required to confirm this observation. A retrospective study of 78 patients in whom investigation was indicated documented endoscopic findings ^34^. The study concluded that investigation was useful to diagnose inflammatory bowel disease and differentiate CGD-associated inflammatory bowel disease from other causes of colitis. Additionally, the severity of disease could be determined, and response to treatment determined.

An interesting study examined the role of gastrointestinal microbiota in pathogenesis of colitis in p47 ^phox−/−^ mice ^35^, suggesting that the signature of the microbiome played a significant role in resistance to, or development of, dextran sodium sulfate-induced colitis. These data support the concept that intestinal bacteria play a significant role in the development and maintenance of colitis, although human studies are required to confirm this. Intriguingly, haematopoietic stem transplantation to restore p47 ^phox^ function failed to resolve colitis in the mouse model, findings not observed in patients who have been transplanted. Whether this is a species-specific finding or instead suggests non-haematopoietic cell production of ROS needs further exploration.

## Inflammation in chronic granulomatous disease

A number of important studies have recently been published which add information to our understanding of the development of inflammatory complications in CGD. Patients experience symptoms, which overlap with those experienced by patients with systemic lupus erythematosus (SLE). Neutrophil-derived proteolytic enzymes and reactive intermediate oxygen species are implicated in inflammatory responses through interaction with innate and adaptive immune pathways. Neutrophil extracellular traps (NETs) are DNA structures formed because of chromatin decondensation and spreading. Numerous proteins that have bactericidal activity and are able to destroy virulence factors, including histones, elastase, myeloperoxidase, cathepsin G, and lactoferrin, adhere to NETs. Formation of NETs is implicated in autoimmunity through opsonisation by autoantibodies leading to plasmacytoid dendritic cell–induced interferon-alpha (IFN-α) synthesis and inflammasome activation. Ribonucleoprotein-containing immune complexes, which are abundant in SLE, can induce NET formation, as can low-density granulocytes, a pro-inflammatory neutrophil subset present in individuals with SLE. Other inducers of NETs include pathogens and cytokines such as interleukin 1 beta (IL-1β). Production of ROS by NOX-dependent pathways is necessary for NET formation. A study by Lood and colleagues demonstrated the ability of mitochondrial ROS to stimulate NET formation in the pro-inflammatory low-density granulocytes in lupus and CGD subjects ^36^. Additionally, they demonstrated that the release of oxidised mitochondrial DNA with potent pro-inflammatory and IFN-driven properties was able to generate NETs even in CGD patients with reduced or absent functional NOX. This study indicates that a key pathway of innate immune activation predisposing to autoimmunity in patients with CGD is similar to that of patients with SLE. That pathway is mitochondrial ROS-driven NET formation in low-density granulocytes, which stimulates externalisation of pro-inflammatory oxidised mitochondrial DNA with consequent activation of stimulator of IFN genes (STING) and Toll-like receptor (TLR)-dependent type I IFN synthesis.

The importance of these findings is emphasised by a study by Weisser and colleagues, who demonstrated that persistent chronic inflammation in patients with CGD leads to haematopoietic proliferative stress and a decrease in the functional activity of haematopoietic stem cells ^37^. Quality and quantity of haematopoietic stem cells are affected, leading to a low proportion of primitive haematopoietic stem or progenitor cells in bone marrow from patients with X-linked CGD, which has implications for those undergoing autologous gene therapy. Treatment directed at this pathway may reduce the autoimmune predisposition as well as improve haematopoietic stem cell mobilisation for gene therapy.

Macrophage activation syndrome is caused by excessive activation and proliferation of well-differentiated macrophages and is synonymous with haemophagocytic lymphohistiocytosis (HLH), a potentially life-threatening hyper-inflammatory syndrome. Clinical features include persistent fever, lymphadenopathy, hepatosplenomegaly, cytopenia, raised liver cell enzymes, and coagulopathy. Most commonly associated with hereditary HLH syndromes, it has been described in a variety of other primary immunodeficiencies. A recent survey found that over 30% of non-hereditary HLH syndrome cases were associated with CGD ^38^. Overall survival for this sub-group was good, and most received treatment with immunoglobulin and cortico-steroids rather than one of the standard HLH protocols. The failure to identify HLH in other neutrophil disorders may suggest a genetic predisposition in CGD patients, associated with a hyper-inflammatory phenotype, rather than an infectious trigger.

## X-linked carrier disease

Female carriers of X-linked AR disorders are generally considered to be clinically unaffected by the defective gene. In X-linked CGD, female carriers possess a mutated gene on one X chromosome and a normal copy on the other X chromosome and, subsequent to lyonisation, have two phagocyte populations: one which is gp91 ^phox^-positive and one which is gp91 ^phox^-negative. Early in foetal development, random X chromosome inactivation of haematopoietic precursor cells may lead to two unequal populations. It is unclear whether the proportion of normal to affected cells can change over time. For many years, it has been acknowledged that female carriers of X-linked CGD may experience discoid lupus erythematosis-like lesions ^39^. However, it is increasingly evident that carriers may display clinical manifestations similar to those of patients with X-linked CGD, which may significantly impact health. A recent study documented wide-ranging autoimmune and inflammatory symptoms in the UK cohort of 81 X-linked CGD carriers, similar to clinical presentations of CGD patients. Specifically, 25% of carriers met at least four of the American Rheumatology Association SLE diagnostic criteria, although only 18% had been diagnosed with a lupus-like disorder ^40^. Seventy-nine percent of subjects reported skin symptoms, most frequently photosensitivity. Additionally, gastrointestinal symptoms were reported by 49% of carriers, most frequently abdominal pain and diarrhoea, although most did not have a defined diagnosis. Histological features of Crohn’s disease were reported in two carriers, and colitis was reported in one. Colitis in an index CGD case correlated with symptoms in the related carriers. For those carriers who reported joint symptoms, episodic inflammation, pain and redness were common symptoms, associated with extreme fatigue. In a separate study of the same cohort, fatigue was associated with raised serum levels of IL-8 ^41^.

Following this, the NIH published findings on 92 subjects from their carrier cohort ^42^. The percentage of residual respiratory burst was unrelated to age of the subject, but a low percentage of dihydrorhodamine oxidation (median of 8%) correlated with clinical features. In contrast, there was no association of degree of lyonisation with autoimmunity, suggesting that the risk was associated with carrier status alone. Limited data from this study suggested a strong correlation of the degree of lyonisation in sisters but not in mother-daughter pairs.

Hauck and colleagues reported on two unrelated female carriers who had non-random X-chromosome inactivation with neutrophil oxidative bursts of 11% and 30% respectively ^43^. Both developed severe fistulating Crohn’s-like inflammatory bowel disease with histo-pathological features similar to those found in patients with CGD-colitis. The second patient underwent allogeneic haematopoietic stem cell transplantation and had complete remission of bowel symptoms at 16 months of follow-up. A 53-year-old Australian women developed refractory pneumonia from which lobectomy tissue grew *Burkholderia cepacia*. Histopathological examination revealed diffuse pneumonic consolidation with suppurative, necrotising granulomata. An immunological assessment demonstrated reduced neutrophil function due to extreme lyonisation with only 3% normally functioning neutrophils. A pathogenic mutation in *CYBB* was found on sequencing, confirming her status as a symptomatic carrier of X-linked CGD; a positive family history was subsequently elicited ^44^.

These studies are important because they demonstrate that X-linked carriers experience significant symptoms, many of which have been under-recognised by medical professionals and many of which can be addressed. Fatigue is a particular issue, which appears to have an inflammatory basis. Furthermore, some individuals have symptoms as severe as those of CGD patients, often associated with extreme lyonisation, and definitive therapy has a role in these individuals. Finally, they raise doubt about the prudence of using carriers as sibling or parental donors for patients with CGD.

## New treatment strategies

### Anti-inflammatory therapy

Inflammatory complications, particularly colitis, are amongst the most challenging symptoms to address in patients with CGD, as treatment with immunosuppressive agents might increase the risk of infection ^45^. By recognising the role of pro-inflammatory cytokines such as IL-1β in the inflammatory process, targeted blockade may offer a focused treatment. However, in contra-distinction to a previous report suggesting that treatment with the recombinant IL-1 receptor targeted antagonist, anakinra, offered therapeutic benefit ^46^, treatment of five patients with severe refractory colitis led to marginal or no benefit ^47^. However, despite treatment for at least 3 months, no significant infectious complications were encountered. Further studies are required to determine whether IL-1 receptor blockade offers benefit for any group of patients with CGD.

To pursue that idea further, a more recent study focused on ways to counter the defect in autophagy and associated inflammasome activation evident in NOX defective mononuclear phagocytes ^48^. Interestingly, even patients without inflammatory manifestations had a reliable inflammatory phenotype characterised by an increase in non-classic and intermediate monocytes, a pro-inflammatory state of mononuclear phagocytes with increased IL-1β and tumour necrosis factor alpha (TNFα) content, a bias of CD4 ^+^ T lymphocytes toward a TH17 phenotype, and increased in IL-17A–secreting neutrophils. *In vitro* studies of mononuclear phagocytes from patients with clinical inflammation, treated *in vitro* with the mechanistic target of rapamycin (mTOR) inhibitor, rapamycin, to restore autophagy, showed reduced basal TNFα production and secretion and suppression of IL-1β, IL-6 and IL-23 secretion by lipopolysaccharide-primed monocytes, with impaired TH17 switching ^48^. Of note, inhibition of the IL-1 receptor with anakinra, enhanced the inhibitory effect of rapamycin on phagocyte IL-1β secretion. These *in vitro* data suggest that a combination of rapamycin with anakinra could be considered for patients with CGD with inflammatory complications.

A single case reports the use of the IL-23 antagonist ustekinumab to treat longstanding treatment-recalcitrant colitis ^49^. There was significant symptomatic improvement, but the patient developed probable severe infection necessitating discontinuation of ustekinumab, after which symptoms returned ^49^. More studies are required to define which of these agents, and in which combinations, lead to infection-free clinical benefit.

### Adjunctive therapy

Whilst antimicrobial ^50, 51^ prophylaxis, with immunosuppression as required remains the mandatory treatment for patients with CGD, and in many centres, IFNγ prophylaxis ^52^, nevertheless, adjunct therapies are sometimes required to treat refractory infection.

Pioglitazone is a peroxisome proliferator–activated receptor gamma (PPARγ) agonist used in the treatment of type 2 diabetes. A recent study demonstrates that, in murine models and human cells, pioglitazone enhanced stimulated mitochondrial-derived ROS production in neutrophils and monocytes from blood and neutrophils and inflammatory macrophages recruited to tissues and thus represents a possible new treatment strategy in CGD patients with severe infection ^53^.

The value of granulocyte infusions to treat such infections remains controversial and there are no trial data to demonstrate efficacy, although case reports suggest efficacy in treating serious bacterial and fungal infections ^54^. A retrospective study of 69 infusion courses in 48 patients in a non-transplant setting reports on the outcome over a 29-year period ^55^. More than 80% of infections improved. Best results seen when infusions were started early in the disease course, and when higher numbers of infusions were administered, irrespective of disease genotype, infection site or causative agent. Adverse events—including fever, rigors, flushing, vomiting, irritability, or agitation, and, in two patients, transient dyspnoea—were reported in less than 2% of 1594 individual infusions. Significantly, 16 patients developed allo-immunisation. This study demonstrates that this treatment approach is generally safe and has utility, although sourcing granulocytes can be difficult, and for those patients in whom stem cell transplantation is an option, allo-immunisation may complicate subsequent treatment.

The utility of thoracic surgery in patients with severe pulmonary infection is reported ^56^. The outcome of 51 thoracic procedures in 35 patients over a 25-year period was documented. The 90-day mortality was 6%, and 37% of patients had died by the time of analysis, predominantly from pulmonary infection. Poor prognostic features were chest wall resection or significant intra-operative blood loss. Patients requiring thoracic surgery are high-risk and, following surgery, should be considered for definitive treatment.

### Haematopoietic stem cell transplantation

To date, haematopoietic stem cell transplantation remains the only definitive curative treatment. Numerous studies have shown efficacy, and an improvement in survival, most notably that reported by Güngör and colleagues using a targeted sub-myelo-ablative busulfan conditioning regimen ^57^. Now results of a multi-centre study using a treosulfan stem are reported ^58^. Treosulfan-based regimens are myelo-ablative but associated with a low toxicity profile. Seventy paediatric patients from 16 centres worldwide were transplanted between 2006 and 2015, the majority with features associated with poor transplant outcomes. No major treatment-related toxicities were reported. The overall survival was 91.4%, and event-free survival was 81.4% at a median follow-up of 34 months, and there was a low cumulative incidence of acute grade III-IV graft-versus-host disease (12%). Complete myeloid donor chimerism was documented in 80% of surviving patients. However, secondary graft failure occurred in 12% of patients. This study demonstrates that treosulfan-containing conditioning regimens can be used safely for children with CGD and high-risk clinical features, achieving an excellent survival and high percentage of complete myeloid chimerism. Further studies are required to compare this regimen with others, particularly to evaluate the long-term outcome, particularly regarding durability of myeloid chimerism and fertility.

Novel methods of T-lymphocyte depletion are increasingly used as treatment for patients without HLA-matched stem cell donors. The method most commonly reported to date in patients with primary immunodeficiency uses an organic iron bead attached to an antibody to remove CD3 TCRαβ and CD19 ^+^ lymphocytes *ex vivo* from the other cells by passing the haematopoietic stem cell source through a magnetic column ^59– 61^. From these series, four patients with CGD were transplanted successfully; one rejected the graft, most likely because of inadequate conditioning, but was successfully re-transplanted. High-dose post-transplant cyclophosphamide has been used to treat malignant and non-malignant disease. One patient with X-linked CGD has been successfully treated with this method using a haploidentical donor. Despite active *Scedosporium apiospermum* infection and development of grade 2 graft-versus-host disease, the patient was well at 9 months of follow-up ^62^.

### Gene therapy

Modification of germline DNA to correct mutated genes is a natural treatment progression from replacement of defective recipient haematopoietic stem cells with normal allogeneic donor cells. Initial gene addition trials using gamma-retroviral vectors to treat X-linked CGD achieved limited gene transduction, resulting in low levels of functional protein. Nevertheless, patients were able to clear persistent life-threatening fungal infection and receive significant clinical benefit. However, whilst methylation of the long terminal repeat (LTR) promoter elements led to gene expression silencing and disappearance of functional neutrophils, the intact enhancer activity of the viral vector drove transactivation of proto-oncogenes, leading to myelodysplasia in three patients ^63^. Multi-centre clinical trials using a new self-inactivating lentiviral vector are in progress, but no results have been published to date.

Rapid developments in methods of integrating viral vectors into ‘safe’ genomic regions to reduce risks of ‘off-target’ effects and in methodsof gene editing have been recently reported. Two groups have demonstrated targeted insertion of a gp91 ^phox^ construct into safe genomic regions. An electroporated zinc finger nuclease mRNA and adeno-associated virus 6 delivery of donor constructs into human haematopoietic stem cells demonstrated significant levels of targeted integration into a safe harbour locus ^64^, with almost clinically relevant levels of gp91 ^phox^ expression and increased NOX activity in *in vitro*–derived neutrophils. A second group used transcription activator-like effector nucleases (TALENs) to insert a myelo-specific gp91 ^phox^ cassette into patient-derived induced pluripotent stem cells (iPSCs) and demonstrated restored ROS production from corrected X-CGD iPSC-derived granulocytes that induced NET formation ^65^. Targeted insertion of an exon 2–13 minigene into exon 2 of *CYBB* resulted in normal regulation of gp91 ^phox^ expression by the endogenous *CYBB* promoter and restored gp91 ^phox^ and NOX activity, demonstrating the necessity of retention of intronic elements for expression of the minigene ^66^. CRISPR-Cas9 correction of *CYBB* ^67^ and *NCF1* ^68^ has also been demonstrated recently. Finally, a zinc finger nuclease has been used to correct the GT deletion at the start of exon 2 in an NCF1 pseudogene and demonstrate restoration of function ^69^.

## Conclusions

The last few years have seen tremendous advances in our knowledge of CGD. The problems experienced by X-linked carriers, specifically a lupus-like disorder or other autoimmune manifestations and fatigue, have been over-looked, and many would benefit from focussed medical care. The rapid progress in gene therapy is extremely exciting, and clinical trials using gene-editing techniques are likely to be developed within the next 5 years. In the meantime, haematopoietic stem cell transplantation is safe, even for patients with severe infection or inflammation, and stem cell manipulation techniques should mean that a suitable donor is available for every patient.

## Abbreviations

AR, autosomal recessive; BCG, Bacillus Calmette–Guérin; CGD, chronic granulomatous disease; HLH, haemophagocytic lymphohistiocytosis; IFN, interferon; IL, interleukin; iPSC, induced pluripotent stem cell; NADPH, nicotinamide adenine dinucleotide phosphate; NET, neutrophil extracellular trap; NIH, National Institutes of Health; NOX, nicotinamide adenine dinucleotide phosphate oxidase; ROS, reactive oxygen species; SLE, systemic lupus erythematosus; TNFα, tumour necrosis factor alpha.

## References

[1] Berendes H Bridges RA Good RA: A fatal granulomatosus of childhood: the clinical study of a new syndrome. *Minn Med.* 1957;40(5):309–12. 10.1001/archpedi.1959.02070010389004 13430573

[2] Jones LB McGrogan KR Flood TJ: Special article: chronic granulomatous disease in the United Kingdom and Ireland: a comprehensive national patient-based registry. *Clin Exp Immunol.* 2008;152(2):211–8. 10.1111/j.1365-2249.2008.03644.x 18410635 PMC2384093

[3] Segal BH Leto TL Gallin JI: Genetic, biochemical, and clinical features of chronic granulomatous disease. *Medicine (Baltimore).* 2000;79(3):170–200. 10.1097/00005792-200005000-00004 10844936

[4] Mouy R Fischer A Vilmer E: Incidence, severity, and prevention of infections in chronic granulomatous disease. *J Pediatr.* 1989;114(4 Pt 1):555–60. 10.1016/S0022-3476(89)80693-6 2784499

[5] Ahlin A De Boer M Roos D: Prevalence, genetics and clinical presentation of chronic granulomatous disease in Sweden. *Acta Paediatr.* 1995;84(12):1386–94. 10.1111/j.1651-2227.1995.tb13575.x 8645957

[6] Winkelstein JA Marino MC Johnston RB Jr: Chronic granulomatous disease. Report on a national registry of 368 patients. *Medicine (Baltimore).* 2000;79(3):155–69. 10.1097/00005792-200005000-00003 10844935

[7] Martire B Rondelli R Soresina A: Clinical features, long-term follow-up and outcome of a large cohort of patients with Chronic Granulomatous Disease: an Italian multicenter study. *Clin Immunol.* 2008;126(2):155–64. 10.1016/j.clim.2007.09.008 18037347

[8] van den Berg JM van Koppen E Ahlin A: Chronic granulomatous disease: the European experience. *PLoS One.* 2009;4(4):e5234. 10.1371/journal.pone.0005234 19381301 PMC2668749

[9] Cole T McKendrick F Titman P: Health related quality of life and emotional health in children with chronic granulomatous disease: a comparison of those managed conservatively with those that have undergone haematopoietic stem cell transplant. *J Clin Immunol.* 2013;33(1):8–13. 10.1007/s10875-012-9758-0 23011479

[10] Wu J Wang WF Zhang YD: Clinical Features and Genetic Analysis of 48 Patients with Chronic Granulomatous Disease in a Single Center Study from Shanghai, China (2005-2015): New Studies and a Literature Review. *J Immunol Res.* 2017;2017:8745254. 10.1155/2017/8745254 28251166 PMC5303869

[11] Xu H Tian W Li SJ: Clinical and molecular features of 38 children with chronic granulomatous disease in mainland china. *J Clin Immunol.* 2014;34(6):633–41. 10.1007/s10875-014-0061-0 24943880

[12] Roos D: Comments on J Clin Immunol (2014) 34:633–641 DOI 10.1007/s10875-014-0061-0. Clinical and Molecular Findings of 38 Children with Chronic Granulomatous Disease in Mainland China, by Huan Xu et al. *J Clin Immunol.* 2017;37(1):3–4. 10.1007/s10875-016-0329-7 27582172

[13] Rawat A Vignesh P Sharma A: Infection Profile in Chronic Granulomatous Disease: a 23-Year Experience from a Tertiary Care Center in North India. *J Clin Immunol.* 2017;37(3):319–28. 10.1007/s10875-017-0382-x 28332028

[14] Kulkarni M Desai M Gupta M: Clinical, Immunological, and Molecular Findings of Patients with p47 ^phox^ Defect Chronic Granulomatous Disease (CGD) in Indian Families. *J Clin Immunol.* 2016;36(8):774–84. 10.1007/s10875-016-0333-y 27699571

[15] El Hawary R Meshaal S Deswarte C: Role of Flow Cytometry in the Diagnosis of Chronic Granulomatous Disease: the Egyptian Experience. *J Clin Immunol.* 2016;36(6):610–8. 10.1007/s10875-016-0297-y 27222152

[16] Ben-Farhat K Ben-Mustapha I Ben-Ali M: A Founder Effect of c.257 + 2T > C Mutation in *NCF2* Gene Underlies Severe Chronic Granulomatous Disease in Eleven Patients. *J Clin Immunol.* 2016;36:547–54. 10.1007/s10875-016-0299-9 27220316

[17] Al-Zadjali S Al-Tamemi S Elnour I: Clinical and molecular findings of chronic granulomatous disease in Oman: family studies. *Clin Genet.* 2015;87(2):185–9. 10.1111/cge.12351 24446915

[18] Kim YM Park JE Kim JY: Genetic analysis of 10 unrelated Korean families with p22-phox-deficient chronic granulomatous disease: an unusually identical mutation of the CYBA gene on Jeju Island, Korea. *J Korean Med Sci.* 2009;24(6):1045–50. 10.3346/jkms.2009.24.6.1045 19949658 PMC2775850

[19] de Boer M Tzur S van Leeuwen K: A founder effect for p47 ^phox^ *Trp193Ter* chronic granulomatous disease in Kavkazi Jews. *Blood Cells Mol Dis.* 2015;55(4):320–7. 10.1016/j.bcmd.2015.07.014 26460255

[20] Wolach B Gavrieli R de Boer M: Chronic granulomatous disease: Clinical, functional, molecular, and genetic studies. The Israeli experience with 84 patients. *Am J Hematol.* 2017;92(1):28–36. 10.1002/ajh.24573 27701760

[21] Kuhns DB Alvord WG Heller T: Residual NADPH oxidase and survival in chronic granulomatous disease. *N Engl J Med.* 2010;363(27):2600–10. 10.1056/NEJMoa1007097 21190454 PMC3069846

[22] Lugo Reyes SO Ramirez-Vazquez G Cruz Hernández A: Clinical Features, Non-Infectious Manifestations and Survival Analysis of 161 Children with Primary Immunodeficiency in Mexico: A Single Center Experience Over two Decades. *J Clin Immunol.* 2016;36(1):56–65. 10.1007/s10875-015-0226-5 26707787

[23] de Oliveira-Junior EB Zurro NB Prando C: Clinical and Genotypic Spectrum of Chronic Granulomatous Disease in 71 Latin American Patients: First Report from the LASID Registry. *Pediatr Blood Cancer.* 2015;62(12):2101–7. 10.1002/pbc.25674 26185101

[24] Conti F Lugo-Reyes SO Blancas Galicia L: Mycobacterial disease in patients with chronic granulomatous disease: A retrospective analysis of 71 cases. *J Allergy Clin Immunol.* 2016;138(1):241–248.e3. 10.1016/j.jaci.2015.11.041 26936803

[25] Dunogué B Pilmis B Mahlaoui N: Chronic Granulomatous Disease in Patients Reaching Adulthood: A Nationwide Study in France. *Clin Infect Dis.* 2017;64(6):767–75. 10.1093/cid/ciw837 28362954

[26] Liese JG Jendrossek V Jansson A: Chronic granulomatous disease in adults. *Lancet.* 1996;347(8996):220–3. 10.1016/S0140-6736(96)90403-1 8551880

[27] Salvator H Mahlaoui N Catherinot E: Pulmonary manifestations in adult patients with chronic granulomatous disease. *Eur Respir J.* 2015;45(6):1613–23. 10.1183/09031936.00118414 25614174

[28] Colin de Verdière S Noel E Lozano C: Respiratory Complications Lead to the Diagnosis of Chronic Granulomatous Disease in Two Adult Patients. *J Clin Immunol.* 2017;37(2):113–6. 10.1007/s10875-017-0370-1 28130637

[29] Tabarsi P Mirsaeidi M Karimi S: Lymphocytic bronchiolitis as presenting disorder in an undiagnosed adult patient with chronic granulomatous disease. *Iran J Allergy Asthma Immunol.* 2007;6(4):219–21. 18094446

[30] Klebanoff SJ White LR: Iodination defect in the leukocytes of a patient with chronic granulomatous disease of childhood. *N Engl J Med.* 1969;280(9):460–6. 10.1056/NEJM196902272800902 4974160

[31] Sibley CT Estwick T Zavodni A: Assessment of atherosclerosis in chronic granulomatous disease. *Circulation.* 2014;130(23):2031–9. 10.1161/CIRCULATIONAHA.113.006824 25239440 PMC4258873

[32] Huang C De Ravin SS Paul AR: Genetic Risk for Inflammatory Bowel Disease Is a Determinant of Crohn's Disease Development in Chronic Granulomatous Disease. *Inflamm Bowel Dis.* 2016;22(12):2794–801. 10.1097/MIB.0000000000000966 27861181 PMC5303573

[33] Broides A Sagi O Pinsk V: Subclinical intestinal inflammation in chronic granulomatous disease patients. *Immunol Res.* 2016;64(1):155–9. 10.1007/s12026-015-8733-2 26603166

[34] Khangura SK Kamal N Ho N: Gastrointestinal Features of Chronic Granulomatous Disease Found During Endoscopy. *Clin Gastroenterol Hepatol.* 2016;14(3):395–402.e5. 10.1016/j.cgh.2015.10.030 26545803 PMC5553864

[35] Falcone EL Abusleme L Swamydas M: Colitis susceptibility in p47 ^*phox-/-*^ mice is mediated by the microbiome. *Microbiome.* 2016;4:13. 10.1186/s40168-016-0159-0 27044504 PMC4820915

[36] Lood C Blanco LP Purmalek MM: Neutrophil extracellular traps enriched in oxidized mitochondrial DNA are interferogenic and contribute to lupus-like disease. *Nat Med.* 2016;22(2):146–53. 10.1038/nm.4027 26779811 PMC4742415

[37] Weisser M Demel UM Stein S: Hyperinflammation in patients with chronic granulomatous disease leads to impairment of hematopoietic stem cell functions. *J Allergy Clin Immunol.* 2016;138(1):219–228.e9. 10.1016/j.jaci.2015.11.028 26853280

[38] Bode SF Ammann S Al-Herz W: The syndrome of hemophagocytic lymphohistiocytosis in primary immunodeficiencies: implications for differential diagnosis and pathogenesis. *Haematologica.* 2015;100(7):978–88. 10.3324/haematol.2014.121608 26022711 PMC4486233

[39] Brandrup F Koch C Petri M: Discoid lupus erythematosus-like lesions and stomatitis in female carriers of X-linked chronic granulomatous disease. *Br J Dermatol.* 1981;104(5):495–505. 10.1111/j.1365-2133.1981.tb08163.x 7236510

[40] Battersby AC Braggins H Pearce MS: Inflammatory and autoimmune manifestations in X-linked carriers of chronic granulomatous disease in the United Kingdom. *J Allergy Clin Immunol.* 2017;140(2):628–630.e6, pii: S0091-6749(17)30428-1. 10.1016/j.jaci.2017.02.029 28343844

[41] Battersby AC Martin AJ Tarn J: Raised Serum IL-8 Levels Are Associated with Excessive Fatigue in Female Carriers of X-Linked Chronic Granulomatous Disease in the UK. *J Clin Immunol.* 2017;37(3):279–81. 10.1007/s10875-017-0384-8 28337619

[42] Marciano BE Zerbe CS Falcone EL: X-linked carriers of chronic granulomatous disease: Illness, lyonization, and stability. *J Allergy Clin Immunol.* 2017; pii: S0091-6749(17)30763-7. 10.1016/j.jaci.2017.04.035 28528201

[43] Hauck F Koletzko S Walz C: Diagnostic and Treatment Options for Severe IBD in Female X-CGD Carriers with Non-random X-inactivation. *J Crohns Colitis.* 2016;10(1):112–5. 10.1093/ecco-jcc/jjv186 26464403

[44] Sarwar G de Malmanche T Rassam L: Chronic granulomatous disease presenting as refractory pneumonia in late adulthood. *Respirol Case Rep.* 2015;3(2):54–6. 10.1002/rcr2.99 26090111 PMC4469140

[45] Uzel G Orange JS Poliak N: Complications of tumor necrosis factor-α blockade in chronic granulomatous disease-related colitis. *Clin Infect Dis.* 2010;51(12):1429–34. 10.1086/657308 21058909 PMC3106244

[46] de Luca A Smeekens SP Casagrande A: IL-1 receptor blockade restores autophagy and reduces inflammation in chronic granulomatous disease in mice and in humans. *Proc Natl Acad Sci U S A.* 2014;111(9):3526–31. 10.1073/pnas.1322831111 24550444 PMC3948220

[47] Hahn KJ Ho N Yockey L: Treatment With Anakinra, a Recombinant IL-1 Receptor Antagonist, Unlikely to Induce Lasting Remission in Patients With CGD Colitis. *Am J Gastroenterol.* 2015;110(6):938–9. 10.1038/ajg.2015.135 26052777

[48] Gabrion A Hmitou I Moshous D: Mammalian target of rapamycin inhibition counterbalances the inflammatory status of immune cells in patients with chronic granulomatous disease. *J Allergy Clin Immunol.* 2017;139(5):1641–1649.e6. 10.1016/j.jaci.2016.08.033 27702670

[49] Butte MJ Park KT Lewis DB: Treatment of CGD-associated Colitis with the IL-23 Blocker Ustekinumab. *J Clin Immunol.* 2016;36(7):619–20. 10.1007/s10875-016-0318-x 27465505 PMC5018915

[50] Margolis DM Melnick DA Alling DW: Trimethoprim-sulfamethoxazole prophylaxis in the management of chronic granulomatous disease. *J Infect Dis.* 1990;162(3):723–6. 10.1093/infdis/162.3.723 2117627

[51] Gallin JI Alling DW Malech HL: Itraconazole to prevent fungal infections in chronic granulomatous disease. *N Engl J Med.* 2003;348(24):2416–22. 10.1056/NEJMoa021931 12802027

[52] A controlled trial of interferon gamma to prevent infection in chronic granulomatous disease. The International Chronic Granulomatous Disease Cooperative Study Group. *N Engl J Med.* 1991;324(8):509–16. 10.1056/NEJM199102213240801 1846940

[53] Fernandez-Boyanapalli RF Frasch SC Thomas SM: Pioglitazone restores phagocyte mitochondrial oxidants and bactericidal capacity in chronic granulomatous disease. *J Allergy Clin Immunol.* 2015;135(2):517–527.e12. 10.1016/j.jaci.2014.10.034 25498313 PMC4331116

[54] Ikincioğullari A Dogu F Solaz N: Granulocyte transfusions in children with chronic granulomatous disease and invasive aspergillosis. *Ther Apher Dial.* 2005;9(2):137–41. 10.1111/j.1774-9987.2005.00227.x 15828925

[55] Marciano BE Allen ES Conry-Cantilena C: Granulocyte transfusions in patients with chronic granulomatous disease and refractory infections: The NIH experience. *J Allergy Clin Immunol.* 2017;140(2):622–625, pii: S0091-6749(17)30424-4. 10.1016/j.jaci.2017.02.026 28342916 PMC5546986

[56] Feingold PL Quadri HS Steinberg SM: Thoracic Surgery in Chronic Granulomatous Disease: a 25-Year Single-Institution Experience. *J Clin Immunol.* 2016;36(7):677–83. 10.1007/s10875-016-0319-9 27497975 PMC6329290

[57] Güngör T Teira P Slatter M: Reduced-intensity conditioning and HLA-matched haemopoietic stem-cell transplantation in patients with chronic granulomatous disease: a prospective multicentre study. *Lancet.* 2014;383(9915):436–48. 10.1016/S0140-6736(13)62069-3 24161820

[58] Morillo-Gutierrez B Beier R Rao K: Treosulfan-based conditioning for allogeneic HSCT in children with chronic granulomatous disease: a multicenter experience. *Blood.* 2016;128(3):440–8. 10.1182/blood-2016-03-704015 27216217 PMC4957165

[59] Bertaina A Merli P Rutella S: HLA-haploidentical stem cell transplantation after removal of αβ+ T and B cells in children with nonmalignant disorders. *Blood.* 2014;124(5):822–6. 10.1182/blood-2014-03-563817 24869942

[60] Balashov D Shcherbina A Maschan M: Single-Center Experience of Unrelated and Haploidentical Stem Cell Transplantation with TCRαβ and CD19 Depletion in Children with Primary Immunodeficiency Syndromes. *Biol Blood Marrow Transplant.* 2015;21(11):1955–62. 10.1016/j.bbmt.2015.07.008 26187864

[61] Shah RM Elfeky R Nademi Z: HLA-haploidentical and Mismatched Unrelated Donor Hematopoietic Stem Cell Transplantation after TCRαβ+ and CD19+ cell depletion in Children with Primary Immune Deficiencies: United Kingdom Experience. *J Allergy Clin Immunol.*(in press).

[62] Parta M Hilligoss D Kelly C: Haploidentical Hematopoietic Cell Transplantation with Post-Transplant Cyclophosphamide in a Patient with Chronic Granulomatous Disease and Active Infection: A First Report. *J Clin Immunol.* 2015;35(7):675–80. 10.1007/s10875-015-0204-y 26453586 PMC6317348

[63] Ott MG Schmidt M Schwarzwaelder K: Correction of X-linked chronic granulomatous disease by gene therapy, augmented by insertional activation of *MDS1-EVI1*, *PRDM16* or *SETBP1*. *Nat Med.* 2006;12(4):401–9. 10.1038/nm1393 16582916

[64] De Ravin SS Reik A Liu PQ: Targeted gene addition in human CD34 ^+^ hematopoietic cells for correction of X-linked chronic granulomatous disease. *Nat Biotechnol.* 2016;34(4):424–9. 10.1038/nbt.3513 26950749 PMC4824656

[65] Dreyer AK Hoffmann D Lachmann N: TALEN-mediated functional correction of X-linked chronic granulomatous disease in patient-derived induced pluripotent stem cells. *Biomaterials.* 2015;69:191–200. 10.1016/j.biomaterials.2015.07.057 26295532

[66] Sweeney CL Zou J Choi U: Targeted Repair of *CYBB* in X-CGD iPSCs Requires Retention of Intronic Sequences for Expression and Functional Correction. *Mol Ther.* 2017;25(2):321–30. 10.1016/j.ymthe.2016.11.012 28153086 PMC5368476

[67] De Ravin SS Li L Wu X: CRISPR-Cas9 gene repair of hematopoietic stem cells from patients with X-linked chronic granulomatous disease. *Sci Transl Med.* 2017;9(372): pii: eaah3480. 10.1126/scitranslmed.aah3480 28077679

[68] Wrona D Siler U Reichenbach J: CRISPR/Cas9-generated p47 ^*phox*^-deficient cell line for Chronic Granulomatous Disease gene therapy vector development. *Sci Rep.* 2017;7:44187. 10.1038/srep44187 28287132 PMC5347011

[69] Merling RK Kuhns DB Sweeney CL: Gene-edited pseudogene resurrection corrects p47 ^phox^ -deficient chronic granulomatous disease. *Blood Adv.* 2016;1:270–8. 10.1182/bloodadvances.2016001214 PMC5727772 29296942

